# Transcranial Focused Ultrasound to the Right Prefrontal Cortex Improves Mood and Alters Functional Connectivity in Humans

**DOI:** 10.3389/fnhum.2020.00052

**Published:** 2020-02-28

**Authors:** Joseph L. Sanguinetti, Stuart Hameroff, Ezra E. Smith, Tomokazu Sato, Chris M. W. Daft, William J. Tyler, John J. B. Allen

**Affiliations:** ^1^Department of Psychology, University of Arizona, Tucson, AZ, United States; ^2^Center for Consciousness Studies, University of Arizona, Tucson, AZ, United States; ^3^Department of Psychology, The University of New Mexico, Albuquerque, NM, United States; ^4^Department of Anesthesiology, University of Arizona, Tucson, AZ, United States; ^5^New York State Psychiatric Institute, New York, NY, United States; ^6^The Division of Biology and Biological Engineering, California Institute of Technology, Pasadena, CA, United States; ^7^River Sonic Solutions LLC, San Francisco, CA, United States; ^8^School of Biological and Health Systems Engineering, Arizona State University, Tempe, AZ, United States

**Keywords:** transcranial focused ultrasound, neuromodulation, mood, functional connectivity, brain stimulation

## Abstract

Transcranial focused ultrasound (tFUS) is an emerging method for non-invasive neuromodulation akin to transcranial magnetic stimulation (TMS) and transcranial direct current stimulation (tDCS). tFUS offers several advantages over electromagnetic methods including high spatial resolution and the ability to reach deep brain targets. Here we describe two experiments assessing whether tFUS could modulate mood in healthy human volunteers by targeting the right inferior frontal gyrus (rIFG), an area implicated in mood and emotional regulation. In a randomized, placebo-controlled, double-blind study, participants received 30 s of 500 kHz tFUS or a placebo control. Visual Analog Mood Scales (VAMS) assessed mood four times within an hour (baseline and three times after tFUS). Participants who received tFUS reported an overall increase in Global Affect (GA), an aggregate score from the VAMS scale, indicating a positive shift in mood. Experiment 2 examined resting-state functional (FC) connectivity using functional magnetic resonance imaging (fMRI) following 2 min of 500 kHz tFUS at the rIFG. As in Experiment 1, tFUS enhanced self-reported mood states and also decreased FC in resting state networks related to emotion and mood regulation. These results suggest that tFUS can be used to modulate mood and emotional regulation networks in the prefrontal cortex.

## Introduction

Transcranial focused ultrasound (tFUS) is an emerging tool for non-invasive neuromodulation that transmits low-intensity ultrasound through the skull to temporarily and safely modulate regional brain activity (Tyler, 2011). Ultrasound neuromodulation offers advantages over transcranial magnetic stimulation (TMS) and transcranial direct current stimulation (tDCS), such as better spatial resolution and the ability to reach deep targets in the brain (Fini and Tyler, 2017). tFUS reversibly modulates neuronal activity in rats (Tufail et al., 2010; Kim et al., 2014), sheep (Lee et al., 2016c), pigs (Dallapiazza et al., 2017), and monkeys (Downs et al., 2016). In humans, tFUS has temporarily altered activity in somatosensory (Lee et al., 2016a), visual (Lee et al., 2016b), and thalamic brain regions (Legon et al., 2018). Researchers are interested in clinical applications of tFUS, including the treatment of psychiatric and neurological disease (Bystritsky and Korb, 2015; Monti et al., 2016). The current experiments investigated whether tFUS could modulate mood in healthy participants by sonicating a region in the prefrontal cortex implicated in emotional regulation, thereby uncovering a target for future therapeutic interventions (Experiment 1). The second experiment used resting-state functional magnetic resonance imaging (fMRI) to investigate FC changes after sonication of the prefrontal cortex to demonstrate that tFUS modulates brain function in networks related to emotional processing and mood.

The prefrontal cortex plays a vital role in emotion and mood regulation (Phan et al., 2002; Coan and Allen, 2004; Ochsner and Gross, 2005; Price and Drevets, 2012). Hemispheric asymmetries in prefrontal activity are thought to contribute to emotional processing (Coan and Allen, 2004; Davidson, 2004; Craig, 2005), and dysfunctions in these networks are related to mood disorders like depression (Stewart et al., 2014) and bipolar disorder (Kerestes et al., 2012). Higher levels of left frontal activity are correlated with more approach motivation (Phillips et al., 2008) and positive mood (Fitzgerald et al., 2008), whereas higher levels of right frontal activity are associated with more withdrawal motivation, negative mood (Hauptman et al., 2008), and increased risk for anxiety and depression (Rempel-Clower, 2007). Currently, TMS and tDCS interventions target lateralized frontal cortex to enhance emotional control in healthy participants or to treat negative mood states in depression (George et al., 2000) and bipolar disorder (Michael and Erfurth, 2004).

In addition to TMS and tDCS, tFUS shows promise as a neuromodulation technique for altering mood states. In a pilot experiment testing the effects of ultrasound neuromodulation on patients, Hameroff et al. (2013) used a clinical ultrasound device at eight megahertz and found that 15 s of sonication of the prefrontal cortex enhanced mood in chronic pain patients, which lasted up to 40 min. Although this experiment suggests that ultrasound neuromodulation could be useful as a therapeutic tool to modulate mood states, the results must be interpreted with caution due to methodological limitations. First, the researchers delivered ultrasound to the prefrontal cortex contralateral to the side that patients reported the most significant pain. In other words, the location where the ultrasound transducer was placed was not uniform across patients. Second, Hameroff and colleagues used an unfocused ultrasound beam applied to the temporal window of the skull, likely sonicating frontal, temporal, and prefrontal cortices. The lack of control of stimulation location makes it impossible to determine whether the unfocused ultrasound affected mood directly, by stimulating a substrate of mood, or indirectly, by modulating other networks, such as those involved in pain perception (i.e., reducing pain perception may lead to more positive mood states). tFUS can untangle these issues by directly targeting brain regions involved in mood and emotional regulation.

One major advantage of tFUS relative to the other neuromodulation techniques like TMS and tDCS is that tFUS has a higher spatial resolution relative to the others. In tFUS applications, the ultrasound beam can be focused at virtually any depth through the human skull to target distinct cortical areas with millimeter resolution (Kubanek, 2018). Lee et al. (2015) showed that tFUS targeting the primary somatosensory cortex produced sonication-specific tactile sensations and somatosensory evoked potentials. Another study further demonstrated the high spatial specificity of tFUS by targeting the primary or secondary sensory cortices with a dual-transducer apparatus, which elicited tactile sensations correlated with the targeted cortical area (Lee et al., 2016a). Sonication of a sub-region of the thalamus with tFUS modulates somatosensory evoked potentials in healthy volunteers, exhibiting the deep focal ability and superior spatial resolution of tFUS (Legon et al., 2018). These experiments suggest that tFUS offers a unique modality for non-invasive modulation of region-specific brain function, and could be a useful method for exploring the effects of ultrasound neuromodulation on emotional regulation centers in the prefrontal cortex.

The goal of the current study was to use tFUS to modulate mood by targeting the right ventrolateral prefrontal cortex (rVLPFC), one of the major areas in the prefrontal cortex for emotional control and mood regulation (Sang and Hamann, 2007), and in particular the regulation and suppression of negative emotions (Ochsner and Gross, 2005; Goldin et al., 2008; Wager et al., 2008). Increased activity of rVLPFC is associated with less negative emotional experience when participants view aversive stimuli (Wager et al., 2008), and symptoms of depression inversely correlate with rVLPFC activity (Drevets et al., 2008). Several experiments show that modulation of the rVLPFC can alter the subjective experience of emotions. For instance, the application of anodal tDCS over the rVLPFC reduces negative feelings in social isolation video games (Riva et al., 2012, 2015a,b) and reduces emotional reactions to negative video clips, even when participants are not explicitly told to suppress negative emotion (Vergallito et al., 2018). Thus, the rVLPFC can serve as a target to enhance control over the emotional experience, which may lead to more positive mood states. Given the focal specificity of tFUS, we chose to target a specific region in rVLPFC, the right inferior frontal gyrus (rIFG; BA 35). The rIFG is a central hub for inhibition and cognitive control (Aron et al., 2014) and has been demonstrated to promote control over emotional processing (Chiu et al., 2008). We predicted that tFUS to the rIFG, using pulse parameters previously shown by Hameroff et al. (2013) to modulate mood in chronic pain patients, would enhance mood in healthy volunteers (Experiment 1). Experiment 2 used resting-state fMRI FC analysis to determine whether tFUS to the rIFG temporarily altered networks associated with mood and emotional regulation. These results would support the notion that the rVLPFC (specifically the rIFG) is involved in processing mood states and would serve as the foundation for future research investigating therapeutic applications of tFUS for mood-related disorders.

## Experiment 1

### Methods

#### Participants

The Institutional Review Board of the University of Arizona approved the experimental protocol. From an introductory-level psychology class, 51 volunteers (27 female, mean age 19.7 years) participated and received class credit. All participants signed an informed consent document. Participants had no history of epilepsy, severe neurological problems, or psychiatric history, and were medication free and not pregnant. All participants were right-handed. Participants were randomly assigned to either the tFUS-Active (*n* = 25) or Placebo condition (*n* = 26). We removed data from three participants due to technical malfunction of the computer that collected mood responses, yielding a final sample of 48 participants (tFUS-Active *n* = 24; Placebo *n* = 24).

#### Experimental Design and Procedures

The experiment was conducted in a small private room at the University of Arizona and occurred between 10 AM and 5 PM. During the consent process, we told participants that the purpose of the study was to test the effects of ultrasound on mood, but we did not specify whether they should expect to feel better or worse after sonication (i.e., after receiving ultrasound). The experimenters followed a structured protocol that would minimize the chance that interaction could prejudice the volunteers about mood changes during the consent or experimental procedures. Participants remained seated throughout the experiment. They were instructed to remain seated during the procedures and to respond as honestly and accurately as they could on the mood questionnaires. Participants were not allowed to use any electronic devices (e.g., cellular phones) during the experiment and were asked to sit quietly until they received instructions. The researchers did not engage in conversation with the participants and only answered questions if the participants asked.

After providing consent, the researcher marked the participants head at a location directly above the rVLPFC: the F8 electrode location [International 10/20 EEG placement system; Klem et al. (1999)]. The J&J psychophysiology system recorded the electrocardiogram (ECG), with bipolar electrodes situated under a wrist strap on the arms, and a strap was placed around the upper abdomen, on the outside of the shirt, to measure respiration. Average heart rate, heart-rate variability (HRV), and respiratory sinus arrhythmia (RSA) were derived. These cardiovascular psychophysiological measures (average heart rate, HRV, and RSA) did not vary as a function of tFUS condition and are therefore not discussed further.

Measurements occurred at four-time points within a 1-h time frame: Once during a baseline period before tFUS (Baseline), 10 min after tFUS (Post-10), 20 min after tFUS (Post-20), and 30 min after tFUS (Post-30). At the start of each of the four data recording assessments, participants sat quietly for 5 min (for the ECG baseline), after which they rated their subjective mood states by filling out a Visual Analog Mood Scale (VAMS; Ahearn, 1997; Nyenhuis, 1997) on a computer (Marsh-Richard et al., 2009). The VAMS is composed of eight questions related to mood and arousal. Participants rated their answer on a scale ranging from 0 to 10. The categories were Happy, Calm, Sad, Tense, Alert, Sleepy, Effort, and Weary. From these categories, we calculated a metric for Global Affect (GA; feelings and mood) and another for Global Vigor [GV; alertness and vigilance (Monk, 1989)]. An increase in the GA rating would indicate an overall positive increase in affective state (happiness, calmness, and reverse-keyed sadness and tenseness). An increase in GV would indicate an overall increase in arousal (alert, reverse keyed weariness, effort, and sleepiness). These measures were the primary dependent variables.

The custom ultrasound system had two modes: stimulation and placebo. The researcher entered a unique five-digit code for each session that would select the mode; the experimenter was blinded to code-condition assignments. Stimulation mode emitted the ultrasound parameters outlined below, and the placebo mode emitted no ultrasound. The device had an LED screen with a timer count-down on it to notify the researcher when 10 min had passed. The screen looked the same for stimulation and placebo modes. Therefore, the researchers and participants were blind to the condition. An offsite researcher (TS), who had no contact with the participants or experimenters, created the randomization codes.

#### Safety

The use of ultrasound, or any source of energy on tissue, requires consideration of significant bioeffects. The effects of ultrasound on living tissue have been well-studied (Dalecki, 2004; O’Brien, 2007; ter Haar, 2007; Church et al., 2008). High-intensity ultrasound can cause tissue heating and cavitation, or small potentially damaging bubbles (usually > 600 W/cm^2^; Wu and Nyborg, 2008). In order to avoid deleterious effects on tissue, the FDA guidelines specify that global maximum acoustic output of ultrasound should be below 720 mW/cm^2^, measured as spatial peak temporal average (*I*_spta_), and a peak average of 190 mW/cm^2^, measured as spatial peak pulse average (*I*_sppa_; Barnett et al., 2000). Decades of animal and human research, as well as thousands of hours of incident-free clinical use, provide evidence that ultrasound at these levels is safe and biological effects are reversible, including effects on the human brain (Tufail et al., 2010; Yoo et al., 2011; Mueller et al., 2014; Downs et al., 2016; Legon et al., 2018, 2012).

#### Focused Ultrasound Waveform

A custom focused ultrasound system generated ultrasound pulses (Neurotrek, Inc., Boston, MA, United States) emitted with a single element transducer (500 kHz, with a two-part lens focused at 30 mm; Blatek, Inc., Pittsburg, PA, United States). The resultant tFUS waveform had the following characteristics: acoustic frequency was 0.5 MHz, pulse duration was 65 μs, pulse repetition period was 23 ms, pulse repetition frequency was 40 Hz, duty cycle was 0.26%, and stimulus duration was 30 s. We chose these parameters on the waveforms used previously to enhance mood states in chronic pain patients (Hameroff et al., 2013) with a diagnostic ultrasound system. The parameters were matched as best as possible given the differences between a single-element focused custom ultrasound system and a phase-array diagnostic ultrasound system. A calibrated hydrophone measured the acoustic intensity (Onda, HN-500, Sunnyvale, CA, United States) by mounting the hydrophone on a three-axis stage positioning system and submerging the ultrasound transducer in a water tank with degassed water. At the center of the emitted ultrasound beam, the peak rarefactional pressure was 1.27 MPa, the mechanical index was 1.79, *I*_sppa_ was 54 W/cm^2^, and *I*_spta_ was 130 mW/cm^2^. Note, these measurements were taken in water alone (without skull) and therefore represented the energy delivered before transcutaneous and transcranial attenuation. All intensity levels were well below FDA guidelines. When measuring single-channel 500 kHz ultrasound beam characteristics in free water through a human skull, about 70–80% of the intensity is absorbed (Legon et al., 2018). Thus, *I*_sppa_ delivered to brain tissue, attenuated by the skull, would be unlikely to exceed 16.2 W/cm^2^.

**FIGURE 1:**
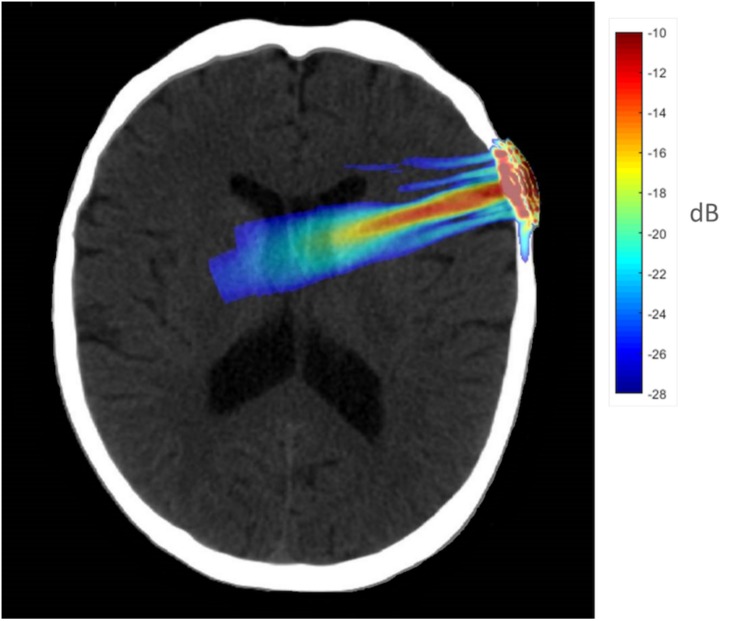
Acoustic simulation model on a representative CT scan of a male patient. The transducer was positioned over the F8 EEG electrode location on the scalp which centers over the rIFG. Hotter colors (red, yellow) indicate more intense sonication, cooler colors (blue, green) indicate less intense sonication.

To better understand the tFUS properties of the focal beam, a head model was created using the K-Wave (Treeby and Cox, 2010) toolbox in MATLAB. A CT scan (randomly chosen from the R.I.R.E. project)^1^ was used to construct the acoustic model of the head. The ultrasound field reported above was entered into the model and projected into the brain assuming the transducer was placed perpendicular to the scalp over F8 (the EEG location centered over the rIFG). The speed of sound entered was 1,550 m/s, and the brain density was 1030 kg/m^3^. Acoustic simulations were performed with the k-Wave MATLAB toolbox on an archival CT scan to estimate the effects of an individual skull on the ultrasound beam properties targeting the rIFG. Figure 1 displays the simulated ultrasound wave propagation through the skull at the location of F8. In this model, the skull reduced the intracranial max acoustic pressure by 53%.

#### Post-experiment Questions

At the end of the experiment, the blinded experimenter queried the participants about their subjective sensations when the transducer was on their head. They were asked to report any sounds they heard from the transducer on their head and were also asked to report any sensations they felt while the transducer was on their head.

#### Statistical Analysis

The dependent variables were GA and GV for the VAMS. We conducted a 4 × 2 repeated measures ANOVA for GA, and separately for GV, with Time of Assessment (Baseline, Post-10, Post-20, and Post-30 assessments) as the within-subjects’ variable and Stimulation (tFUS-Active; Placebo) as the between subjects. Planned comparisons were performed when appropriate.

### Results

#### Post-experiment Questions

A total of 15 out of 24 participants in the tFUS-Active condition reporting hearing some form of “buzzing, clicking or vibrating” when the transducer was on their head, and zero of 24 participants in the Placebo condition reported hearing any form or sound from the transducer. Additionally, 10 of 24 participants in the tFUS-Active condition reported feeling some sensation from the transducer (3 “pulsing,” 5 “buzzing,” and 2 “pressure”); 7 out of 24 participants in the Placebo condition reported feeling some sensation from the transducer (3 “pulsing,” 3 “pressure,” and 1 “warm”). We analyzed the difference in mood reports for participants in the tFUS-Active condition who reported hearing a sound to those who did not (“Sound Report”) to ensure that hearing a sound did not influence the results below. Mauchly’s Test of Sphericity indicated that the assumption of sphericity was violated, χ^2^(5) = 16.112, *p* = 0.007, and thus Greenhouse–Geisser corrections were used, with the original degrees of freedom reported. There was a significant main effect of Time, *F*(3, 66) = 7.536, *p* = 0.002, and there was not a significant interaction between Time of Assessment and Sound Report, *F*(3, 66) = 0.591, *p* = 0.556, indicating that hearing the sound from the transducer did not influence mood reports.

#### Visual Analog Mood Scales

For GA, Mauchly’s Test of Sphericity indicated that the assumption of sphericity was violated, χ^2^(2) = 19.220, *p* = 0.002, and thus Greenhouse–Geisser corrections were used, with the original degrees of freedom reported. There was a significant main effect of Time, *F*(3, 138) = 4.208, *p* = 0.013, $\eta_{ð}^{2}$ = 0.084. There was a significant interaction between Stimulation and Time of Assessment, *F*(3, 138) = 3.817, *p* = 0.019, η_*ð*_^2^ = 0.077. Pairwise comparisons were used to compare each Time of Assessment time-point relative to Baseline (within stimulation conditions; Bonferroni corrected). For participants receiving tFUS-Active, relative to Baseline (*M* = 67.22; SD = 13.74), GA was not significantly higher at the Post-10 condition (*M* = 71.97; SD = 12.05), *p* = 0.173, but was significant at Post-20 (*M* = 75.36; SD = 11.71), *p* = 0.014, and Post-30 (*M* = 75.49; SD = 10.99), *p* = 0.006 (Table 1). No time points differed from baseline for those in the Placebo condition. Individual participant scores for GA and scores for the questions that comprise GA can be found in the Supplementary Material.

**TABLE 1:** Global Affect scores for Experiment 1.

	**Global Affect**			
	**Baseline**	**Post-10**	**Post-20**	**Post-30**
**tFUS-Active**				
Mean	67.22	71.97	75.36*	75.49*
SD	13.74	12.05	11.71	10.99
**Placebo**				
Mean	70.79	67.70	70.28	71.16
SD	13.16	16.00	13.60	11.99

*Significant differences (*p* < 0.05) relative to Baseline are indicated by an asterisk.*

There was no difference in the baseline GA scores between tFUS-Active and Placebo conditions, *p* > 0.05, demonstrating that the groups did not differ in mood reports at the beginning of the experiment.

For GV, Mauchly’s Test of Sphericity again indicated that the assumption of sphericity was violated, χ^2^(2) = 33.915, p = 0.001, and thus Greenhouse–Geisser corrections were used, with the original degrees of freedom reported. There was a main effect of time, *F*(3, 138) = 8.794, *p* < 0.001, η_*ð*_^2^ = 0.160, as participants’ tended to increase in GV over the experiment (Table 2). There was not a significant interaction between Stimulation and Time of Assessment, *F*(3, 138) = 0.620, *p* = 0.537, η_*ð*_^2^ = 0.013 (Table 2).

**TABLE 2:** Global Vigor scores for Experiment 1.

	**Global Vigor**			
	**Baseline**	**Post-10**	**Post-20**	**Post-30**
**tFUS-Active**				
Mean	47.57	47.35	55.41	57.08
SD	14.27	12.57	16.74	17.72
**Placebo**				
Mean	53.44	48.53	56.7	57.89
SD	14.33	16.64	16.93	16.74

*No significant differences were found relative to Baseline.*

The results from Experiment 1 suggest that 30-s exposures of 500 kHz focused ultrasound targeting the rIFG can induce positive mood effects for up to 30 min. Next, we determined the extent to which tFUS modulated brain activity using fMRI.

## Experiment 2

To determine whether tFUS altered brain activity, we recorded resting state fMRI before and 20 min after sonification with the same custom focused ultrasound system from Experiment 1. Resting-state FC analysis was conducted by seeding the rIFG to determine whether sonification altered connectivity patterns relative in the rIFG network. We also seeded major hubs in the Default Mode Network (DMN). The DMN is a highly interconnected network of brain areas that are active when participants are not focused on a task and are instead daydreaming or involved with self-referential processing. Researchers have proposed that the DMN is a fundamental part of the neuronal substrate of the self (Gusnard et al., 2001). In mood disorders like depression, there is enhanced activity in the rIFG and hyper-connectivity in the DMN, which reflects the internal, ruminative nature of depression and the inability to regulate self-referential processes and emotion (Sheline et al., 2009; Kaiser et al., 2016). We hypothesized that 2 min of tFUS to the rIFG would alter connectivity patterns in the rIFG network. Additionally, we predicted that rIFG tFUS would alter connectivity in the DMN in a direction opposite to those patterns found in mood disorders. These results suggest that rIFG enhanced regulation of networks related to emotional processing (rIFG) and self-referential activity (DMN) may lead to altered mood states.

### Methods

#### Participants

The Institutional Review Board of the University of Arizona approved the experiment. Nine volunteers (four females, mean age 19.2) participated. All participants signed an informed consent document. Participants had no history of epilepsy, severe neurological problems, or psychiatric history, and were medication free and not pregnant. All participants were right-handed.

#### Experimental Design and Procedures

After informed consent, participants filled out the VAMS scales (Baseline). Then, we collected 8 min of resting-state neuroimaging data. Participants were not given a task but were told to sit in the MRI scanner with their eyes open. Participants were then taken out of the MRI scanner and immediately received tFUS to the rIFG; they then sat quietly, without interacting with the researchers or anybody else for 10 min before completing the VAMS again; they sat for another 10 min before going back into the MRI scanner (20 min after sonication). After the scan, participants completed the VAMS scales outside the scanner (30 min after sonication). There were only three-time points for VAMS ratings: baseline, 10 min after sonication (Post-10), and 30 min after sonication (Post-30). There was no control condition. For the VAMS scale, we performed an ANOVA with Time as the factor with *post hoc* tests when appropriate. We used the same tFUS device and waveforms from Experiment 1, except the duty cycle increased to 0.5%, and the duration was 2 min. At the center of the emitted ultrasound beam, the peak rarefactional pressure, measured in water, was 1.26 MPa, the mechanical index was 1.79, *I*_sppa_ was 54 W/cm^2^, and *I*_spta_ was 272 mW/cm^2^.

#### fMRI Data Acquisition and Analysis

Functional images were acquired on a Siemens Skyra 3-Tesla scanner using EPI gradient echo sequence (TR = 1800 ms; TE = 25 ms; flip angle = 90; FOV = 192 mm; acquisition voxel size 3 mm × 3 mm × 3 mm). T1-weighted anatomical images were also acquired for registration of the functional scans (MP-RAGE; TR was 2500 ms; TE was 4.35 ms; TI was 900 ms; flip angle was 8; FOV was 256 mm). Data pre-processing and analysis were performed using SPM8 (Welcome Trust Centre for Neuroimaging, University College London, United Kingdom) and the Functional Connectivity Toolbox (CONN; Whitfield-Gabrieli and Nieto-Castanon, 2012) in MATLAB (The MathWorks Inc., United States). Functional volumes underwent realignment and unwarping, slice-timing correction, structural segmentation, functional normalization, outlier detection, spatial smoothing (8 mm full width half maximum Gaussian kernel filter) and were normalized to the Montreal Neurological Institute (MNI) space using the normalized EPI template image (SPM) using CONN’s “defaultMNI” pre-processing pipeline. Noise correction was performed in the CONN toolbox with the CompCor method (Behzadi et al., 2007).

We placed a seed at the tFUS target region (rIFG; BA 45; 58, 13, 6) to determine if network level changes occurred after sonication. We used the 10–20 International EEG coordinate system to place the transducer on the scalp at electrode location F8. We chose BA 45 as our RIO for the rIFG because F8 is correlated with BA 45 and is the most likely region sonicated with a transducer placed on F8 (Koessler et al., 2009). We also examined network-level changes in the DMN after sonication by placing seeds in the medial prefrontal cortex (BA10; 0, 48, −4) and posterior cingulate cortex (BA 31; −5, −51, 39). The DMN BA areas were chosen from the literature suggesting a link between those areas and mood disorder (increased DMN connectivity; e.g., Chen et al., 2015), mind wandering or mindfulness training (reduced DMN connectivity; e.g., Taylor et al., 2013). The seed-to-voxel analysis determined the connectivity of the specific seed regions outlined above with the whole brain and was carried out in CONN. White matter WM, cerebrospinal fluid, realignment parameters, motion artifacts, and physiological noise were taken as confounds and regressed out as implemented with the CompCor strategy (Behzadi et al., 2007). Heart rate and motion artifacts were taken as confounders. The whole-brain BOLD signal was also excluded to eliminate erroneous anti-correlations; the resulting data were bandpass filtered at 0.001 to 0.1 Hz. The temporal correlation between the BOLD signal from a given voxel to all other voxels in the brain was computed.

We computed differences in FC between networks before (pre) and after (post) tFUS with t-tests and Fisher’s Z-transformed correlations in the second-level analysis. A first-level analysis used a general linear model (GLM) to determine significant resting-state connections at the individual level. We reported seed-to-voxel results with significant voxel wise thresholds exceeded at a level of *p* < 0.001 (uncorrected) and a cluster-level threshold of *p* < 0.05 FDR (corrected). Significant clusters (>10 voxels) are reported below.

### Results

#### Visual Analog Mood Scales

For GA, Mauchly’s Test of Sphericity indicated that the assumption of sphericity was violated, χ^2^(2) = 8.891, *p* = 0.012, and thus Greenhouse–Geisser corrections were used, with the original degrees of freedom reported. There was a main effect of time, *F*(2, 16) = 4.908, *p* = 0.049, η_*ð*_^2^ = 0.54. GA ratings did not differ significantly between Baseline (*M* = 81.44, SD = 16.34) relative to Post-10 (*M* = 84.44; SD = 15.45), *p* = 0.31; however, mood significantly improved 30 min after stimulation, Post-30 (*M* = 87.56, SD = 14.89) relative to Baseline, *p* = 0.044 (Table 3).

**TABLE 3:** Global Affect and Global Vigor scores for Experiment 2.

	**Baseline**	**Post-10**	**Post-30**
**Global Affect**			
Mean	81.44	84.44	87.56*
SD	16.34	15.45	14.89
**Global Vigor**			
Mean	75.83	78.06	82.5*
SD	13.22	13.56	13.57

*Significant differences relative to Baseline are indicated by an asterisk. tFUS was active in both conditions.*

For GV, the assumption of sphericity was not violated, χ^2^(2) = 2.605, *p* = 0.272. There was a main effect of time, *F*(2, 16) = 5.439, *p* = 0.016, η_*ð*_^2^ = 0.76. On the GV scale, participants reported the same level of overall mental energy on baseline relative to Post-10, *p* = 0.085; however, participants reported an overall significant increase in mental vigor 30 min after sonication relative to baseline, *p* = 0.028 (Table 3).

#### fMRI Connectivity Results

**FIGURE 2:**
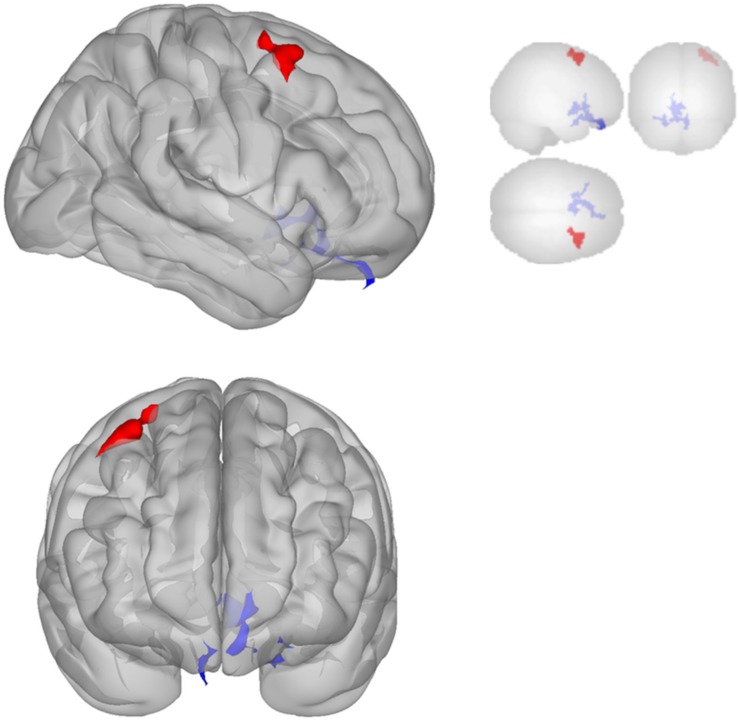
Significant clusters for the rIFG seed-to-voxel analysis. Increased connectivity with rIFG is shown in red and decreased connectivity with rIFG in blue in the Post-scan relative to Baseline.

Functional connectivity decreased after sonication within the rIFG network used in the seed-to-voxel analysis. Compared to the baseline, participants had significantly reduced connectivity between the rIFG and the subgenual cortex, orbitofrontal cortex, inferior prefrontal gyrus, dorsal anterior cingulate cortex, and entorhinal cortex (Table 4 and Figure 2). The analysis revealed significant increases in connectivity between the rIFG and the premotor cortex (Table 5 and Figure 2).

**TABLE 4:** Seed-to-voxel connectivity values for each seed region.

**Reduced functional connectivity post-relative to pre for three seed regions**							
**Seed region**	**Cluster coordinates**	**Cluster size**	**Cluster regions**	**BA**	**Voxels in regions**	**Coverage (%)**	**Cluster p value (*p* < 0.05 FDR)**
Inferior Frontal Gyrus	−06 + 28 − 24	548	(L) Subgenual cortex	25	101	17	0.001
			(R) Orbitofrontal cortex	11	83	3	
			(L) Inferior prefrontal gyrus	47	41	2	
			(L) Orbitofrontal cortex	11	32	1	
			(L) Dorsal anterior cingulate	32	17	1	
			(L) Posterior entorhinal cortex	28	12	2	
			(L) Anterior entorhinal cortex	34	12	2	
			(R) Subgenual cortex	25	4	1	
			Not assigned or <1% coverage		246		
Medial Prefrontal	−12 + 08 + 48	232	(L) Premotor cortex	6	96	1	0.008
			(L) Ventral anterior cingulate	24	66	4	
			(R) Premotor cortex	6	45	1	
			Not assigned or <1% coverage	–	25	–	
Posterior Cingulate	+20 − 40 − 10	263	(R) Parahippocampal cortex	36	97	13	0.002
			(R) Fusiform gyrus	37	47	3	
			(R) Associative visual cortex	19	26	1	
			(R) Perirhinal Cortex	35	18	5	
			(R) Posterior entorhinal cortex	28	7	1	
			Not assigned or <1% coverage	–	68	–	
	−34 − 88 + 28	145	(L) Associative visual cortex	19	105	2	0.033
			Not assigned or <1% coverage	–	40	–	

*Reduced connectivity values are shown in this table, for the post-sonication scan relative to baseline.*

**TABLE 5:** Seed-to-voxel connectivity values for each seed region.

**Increased functional connectivity post-relative to pre by seed region**							
**Seed region**	**Cluster coordinates**	**Cluster size**	**Cluster regions**	**BA**	**Voxels in region**	**Coverage (%)**	**Cluster p value (*p* < 0.05 FDR)**
Inferior Frontal Gyrus (BA 44)	+ 40 + 08 + 60	300	(R) Premotor cortex	6	253	3	0.001
			Not assigned or <1% coverage	–	47	–	
Medial Prefrontal (BA 10)	+ 52 − 6 − 4	220	(R) Superior temporal gyrus	22	98	4	0.008
			(R) Insular cortex	13	41	2	
			(R) Primary auditory cortex	41	39	6	
			(R) Subcentral area	43	12	4	
			Not assigned or <1% coverage	–	30	–	

*Increased connectivity values are shown in this table, for the post-sonication scan relative to baseline.*

**FIGURE 3:**
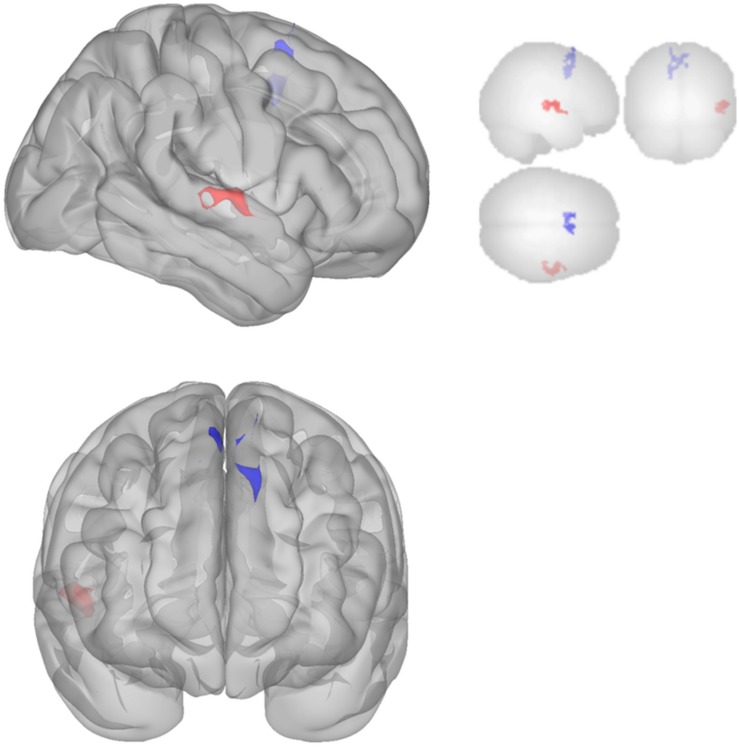
Significant clusters for the medial prefrontal cortex (MPFC) seed-to-voxel analysis. Increased connectivity with MPFC is shown in red and decreased connectivity with MPFC in blue in the Post-scan relative to Baseline.

**FIGURE 4:**
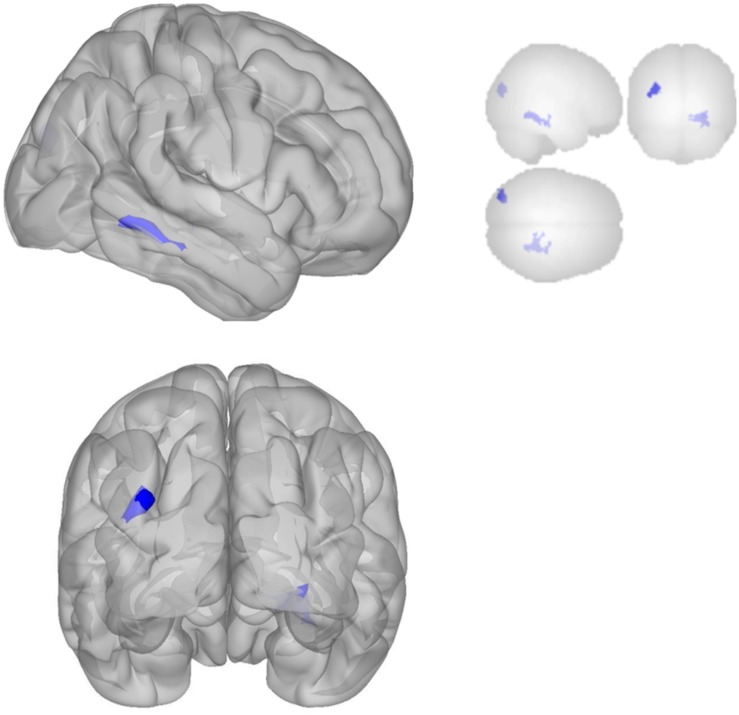
Significant clusters for the medial posterior cingulate gyrus seed-to-voxel analysis. Increased connectivity with posterior cingulate gyrus is shown in red and decreased connectivity with posterior cingulate gyrus in blue in the Post-scan relative to Baseline.

The DMN demonstrated decreased connectivity after sonication. For the MPFC seed, there was decreased connectivity with the premotor cortex, and ventral anterior cingulate cortex (Table 4 and Figure 3) and increased connectivity with the superior temporal gyrus, insular cortex, primary auditory cortex, and subcentral area (Table 5 and Figure 3). The PCC seed demonstrated decreased connectivity with the parahippocampal cortex, fusiform gyrus, perirhinal cortex, entorhinal cortex, and associative visual cortex (Table 4 and Figure 4). Figures 2, 3 represent the regions of interest (seeds) and the corresponding locations of clusters of significant difference between pre- and post-sessions.

The results from Experiment 2 show that 2 min of tFUS targeting the rIFG modulated FC in a network related to the rIFG as well as the DMN 20 min after sonication. These results suggest that tFUS has effects on brain networks related to the area of sonication that lasts up to 20 min.

## General Discussion

Here we report two experiments that demonstrate for the first time that tFUS targeting the rIFG enhances mood, accompanied by changes in FC in networks related to emotional regulation. In a double-blind, placebo-controlled experiment, participants reported a significant increase in mood 20 and 30 min after tFUS (Experiment 1). Experiment 2 replicated the positive mood effects of rIFG sonication and demonstrated FC changes in the rIFG network and the DMN after tFUS. Overall, we found an increase in connectivity between the rIFG and right middle frontal gyrus (rMFG) and decreased connectivity with left prefrontal and limbic areas. Regions within the DMN showed a general decrease in FC.

Previous research with ultrasound has demonstrated that ultrasound can modulate neural activity (see Introduction). Hameroff et al. (2013) found that a diagnostic ultrasound system altered mood in a population of chronic pain patients. We report, for the first time, that tFUS alters mood in healthy participants independent of clinical symptoms. The Hameroff et al. (2013) experiment did not control the location of stimulation, and the participants were chronic pain patients, some of whom were clinically depressed, complicating the interpretation of the result. Nonetheless, Hameroff et al. (2013) induced positive mood changes with 8 MHz stimulation (relative to placebo). In the Hameroff et al. (2013) study, patients reported a slight (but non-significant) decrease in pain. Thus, it is not clear if the elevation in mood was related to the reduction in pain in some participants, from ultrasound altering “mood circuits” in the brain, or a combination of both. By targeting the rIFG in a population of healthy participants with no clinical history or neurological or psychiatric disease, the current experiment is thus the first to demonstrate that tFUS of the rIFG can modulate mood.

The finding that tFUS focused on the rIFG resulted in improved self-reported mood can be understood in terms of the functions and connectivity of the rIFG. The rIFG plays a significant role in response inhibition and executive control (Aron et al., 2014). The rIFG is also involved in exerting cognitive control over emotion networks. For example, when participants are asked to voluntarily inhibit negative emotion during a task, or down-regulate emotion, rIFG is involved (Goldin et al., 2008) as it is generally with regulation of negative emotions (Ochsner and Gross, 2005; Sang and Hamann, 2007; Wager et al., 2008; Berkman and Lieberman, 2009; Parvaz et al., 2012; Touroutoglou et al., 2014). tDCS experiments targeting the same location we targeted in the current experiments have shown that modulation of that region enhances control over emotional experience, especially negative emotions (Riva et al., 2012, 2015b; Vergallito et al., 2018). Along with the middle frontal gyrus and limbic brain regions, the rIFG is part of an important mood regulation network that is related to mood disorders (Phillips et al., 2003). Patients with significant mood symptoms, including those with Parkinson’s Disease, Bipolar Disorder, and Major Depressive Disorder (MDD) have altered connectivity in the rIFG network (Phillips et al., 2008). The results reported here support the notion that the rIFG is involved in a critical network that facilitates the overall regulation of mood states and is a promising target for therapeutic neuromodulation.

Experiment 2 found a significant increase in connectivity between the rIFG and the right middle frontal gyrus (rMFG) after sonication, which may have enhanced participants’ ability to regulate emotional experience and mood during the experiment. Supporting the notion that the rIFG is involved in emotional regulation, research finds that the rIFG is hypoactivated in patients with mood disorders and increases activation after psychotherapy (Fitzgerald et al., 2008). The dorsolateral prefrontal cortex (DLPFC), in the MFG, has been linked to emotional regulation as well (Golkar et al., 2012). Female patients at high risk to develop MDD display decreased connectivity between the rIFG and the rMFG (Clasen et al., 2014). The rIFG-rMFG connectivity increases are suggestive of enhanced inter-region communication after sonication that may enable better regulation of emotional response to the challenges of the experimental setting.

There was also evidence of reduced connectivity between rIFG and left prefrontal and limbic areas. Indeed, left subgenual cortex (BA 25), dorsal anterior cingulate (BA 32), anterior entorhinal cortex (BA 34), left orbitofrontal cortex (BA 11), and left inferior prefrontal gyrus (BA 47), all showed decreased connectivity with the rIFG after sonication. These regions have demonstrated associations with affect and mood. The subgenual cortex (Lozano et al., 2010) is consistently associated with negative affect (Lindquist et al., 2016), and is a primary target of Deep Brain Stimulation for refractory depression. Dorsal anterior cingulate is involved in emotional reappraisal (Kalisch, 2009). The left orbitofrontal cortex (Rempel-Clower, 2007) and left inferior prefrontal gyrus (Ray and Zald, 2012) are involved in emotional regulation. Portions of the limbic system, including the entorhinal cortex, may be involved in mood disorders (Price and Drevets, 2012). The reduced connectivity of these many regions with the rIFG and increased connectivity of rIFG with rMFG, therefore, suggest a re-distribution or re-balancing of activity among a set of brain regions important for emotional experience and regulation.

Changes in connectivity within the DMN were also detected, which may relate to enhanced mood by reducing self-referential thinking and mind-wandering. In particular, the MPFC had reduced connectivity with the ventral anterior cingulate (BA 24), which is a major hub in the DMN (Greicius et al., 2003). The DMN is central to internal, self-referential thinking (Andrews-hanna et al., 2010); hyperconnectivity within the DMN is found for MDD (Kaiser et al., 2015). Alternatively, mindfulness training leads to a decrease in DMN activity, which correlates with positive health outcomes (Keng et al., 2011). Indeed, mind wandering relates to rumination (an essential feature of depression and anxiety), less happiness, and adverse health outcomes (Killingsworth and Gilbert, 2010). Thus, the decreased connectivity in the DMN may indicate a reduction in self-referential thinking and mind wandering, and a state characterized by being engaged in the present moment with the external environment rather than engaging in self-referential processing and rumination, all of which could lead to enhanced mood.

Decreases in the DMN network may also be related to enhanced cognitive control over emotional regulation during the experiment. Relative to the PCC seed, we found decreased connectivity for the right parahippocampal gyrus (BA 36), right temporal fusiform cortex (BA 37), right associative visual cortex (BA 19) and, perirhinal cortex (BA 35). PCC connectivity with parahippocampal gyrus and temporal cortex increases with sad mood induction in depressed patients and decreases in control participants (Renner et al., 2017). The observed changes in the DMN after negative mood induction in depressed patients may reflect an inability to exert cognitive control over emotional processing. Here, the opposite pattern was found, suggesting that the decreased connectivity supported greater cognitive control over emotional states during the experiment, which may have led to enhanced mood.

The effects of tFUS on mood in the current experiments indicate a lag between tFUS exposure and changes in functional brain activity, with effects peaking between 20 and 30 min (Hameroff et al., 2013; Sanguinetti et al., 2013). In experiments on rabbits and felines, respectively, modulatory effects of tFUS on visual evoked potentials lasted for several minutes (Yoo et al., 2011) and 30 min (Fry, 1957). These results suggest that the immediate physiological effects of tFUS may lead to reversible network-level changes over several minutes. The network-level changes could occur through membrane effects but are also consistent with ultrasound having immediate resonant effects on microtubules that result in delayed effects on synaptic plasticity. Assessing the time course of brain activity following tFUS, for example with EEG, could address more specifically how tFUS modulates brain activity and network dynamics, and how these, in turn, relate to mood and mental states.

The mechanisms by which tFUS modulates neuronal activity remains unknown, as is the mechanism by which neuronal activity results in phenomenal experience including mood. Several authors have proposed a mechanosensitivity hypothesis whereby ultrasound affects stretch-sensitive ion channels (Chapman et al., 1980), or lipid membranes surrounding them (Krasovitski et al., 2011), thus affecting membrane conductance (Tyler, 2011; Sassaroli and Vykhodtseva, 2016). Tyler (2011) suggested a “continuum mechanics hypothesis” in which ultrasound alters neuronal excitability through a combination of pressure/fluid/membrane actions involving stable cavitation, acoustic streaming, and fluid dynamics (radiation forces, shear stress, Bernoulli effects). Hameroff and others proposed that tFUS directly affects cytoskeletal microtubules inside neurons (and glia) (Hameroff et al., 2013) which may alter synaptic activity and function and lead to functional changes in brain processes (Hameroff and Penrose, 2014). Indeed, microtubules have been shown to have alternating current (AC) electron conductance resonances in the megahertz (Sahu et al., 2013) range. However, the mechanisms by which tFUS affects neural activity remain unknown, and more research is needed.

### Limitations

The majority of recent human tFUS experiments have focused the sonication beam with MRI-based neuronavigation, which was unavailable for the present studies. Accordingly, we chose to use the 10–20 EEG system to aim the single-element transducer with a 30 mm focal depth beam. The 10–20 EEG system is considered accurate to 0.5-cm resolution (Chatrian et al., 2018) and our focal beam is on the order of millimeters; thus, we cannot validate precise targeting. With this limitation in mind, the experiments reported here demonstrate that tFUS navigated by EEG coordinates is useful to modulate mood in healthy volunteers. Indeed, Legon et al. (2014) showed high spatial specificity with tFUS guided by EEG coordinates to stimulate the somatosensory cortex with at least centimeter resolution. These results could be significant for clinical applications where expensive and time-consuming neuronavigation is not feasible. Future experiments should directly compare the reliability of tFUS navigated by EEG coordinates to tFUS navigated by neuroimaging.

Some participants in the tFUS-Active condition reported hearing a sound when the transducer was on their head while none of the participants in the placebo condition reported hearing a sound. While an audible noise is not necessarily a cue for improved mood or active treatment, hearing a sound could have led participants to believe they were in the active condition thereby altering their mood. To rule out this possibility, we analyzed the changes in mood scores for participants in the active condition who reported hearing a sound to those who did not and found no significant difference in the scores. Furthermore, the difference scores for Post-30 (relative to Baseline) were 4.48 (SD = 9.10) for those who heard a sound and 13.99 (SD = 15.31) for those who did not. Although this difference was not significant, *p* = 0.078, this is the opposite pattern to what would be expected if hearing the sound from the transducer were to bias positive mood reports.

Experiments investigating tFUS in rodents have recently found that induced excitability changes in the brain can be, at least partially, due to an indirect effect of auditory stimulation, which was eliminated by removal of the cochlear fluid (Guo et al., 2018). Additionally, Sato et al. (2018) found that temporary chemical deafness could reduce the effects of tFUS on the brain. These studies show that important confounds can lead to brain activation through indirect pathways, but do not negate the notion that tFUS can also influence the brain directly. Experiments with organisms that lack auditory systems, like Xenopus oocyte (the “clawed frog”), show the effects of tFUS on neural activity (Kubanek et al., 2016), and ultrasound also influences neural activity and causes spike trains in slice preparations (Tyler et al., 2008). In humans, tFUS has produced tactile sensations (Lee et al., 2016a) and visual phosphenes (Lee et al., 2016b) with corresponding focal tissue activation that is hard to explain by activation through ascending auditory activation. Future experiments will need to better control unconscious and conscious auditory effects for ultrasound neuromodulation experiments on mood.

### Future Research and Treatment

Overall, results from the experiments reported here and other recent tFUS studies motivate future investigations into the effects of ultrasound on brain function and cognitive disorders. Specifically, future studies should directly assess the impact of tFUS on experience, behavior, and brain network connectivity, with time-varying assessments in a sufficiently large sample to examine the potential mediational role of changes in network connectivity on mood and behavior. The positive findings reported here motivate testing tFUS in clinical populations with negative affect such as depression and anxiety disorders. Offering advantages over other non-invasive methods like TMS and tDCS, tFUS can be focused through the skull with millimeter precision or used in a wide beam to target large cortical areas. tFUS is relatively inexpensive, safe, and painless and can be used in an MRI or with EEG with little minimal signal interference. Additionally, other brain areas implicated in mood and emotional regulation, e.g., deep brain targets accessible until now only with invasive deep brain stimulation, can be targeted with tFUS. Therefore, tFUS holds excellent promise for the treatment of mental and cognitive disorders.

## Conclusion

Transcranial focused ultrasound at 500 kHz targeting the rIFG for 30 s (Experiment 1) and 2 min (Experiment 2) increased self-reported mood in healthy participants as compared to baseline mood. Corresponding connectivity changes in networks relevant for emotion/mood regulation occurred 20 min after sonication in Experiment 2, demonstrating that tFUS could modulate functionally specific brain networks relevant for mood regulation. These results are in line with recent experiments suggesting that tFUS can modulate network connectivity (Folloni et al., 2019; Verhagen et al., 2019). These results are the first to demonstrate that tFUS can affect mood and cortical networks important for mood regulation, with effects that appear on the order of 20 min following tFUS delivery. Our results show that tFUS aimed at rIFG with a single element transducer can modulate prefrontal cortical activity and improve mood. The present findings suggest that tFUS could be a useful tool in the treatment of clinical disorders characterized by negative mood states, like depression and anxiety disorders and future studies are warranted.

## Data Availability Statement

The datasets generated for this study are available on request to the corresponding author.

## Ethics Statement

The studies involving human participants were reviewed and approved by the Human Subjects Protection Program University of Arizona. The patients/participants provided their written informed consent to participate in this study.

## Author Contributions

JS, SH, TS, WT, and JA conceived and planned the experiments. TS created the blinding procedures. JS and ES carried out the experiments. CD planned and carried out the acoustic modeling. JS performed the mood analysis and functional connectivity analysis. JS, SH, ES, TS, WT, and JA contributed to the interpretation of the results. JS took the lead in writing the manuscript. All authors provided critical feedback and helped to shape the research and manuscript.

## Conflict of Interest

The ultrasound device used in these studies was provided by WT, who is an equity holding founder of IST, LLC, a private neurotechnology company, and the inventor of issued and pending international and US patents covering systems and methods for ultrasonic and bioelectronic neuromodulation. CD was a principal of River Sonic Solutions LLC and is currently self-employed as an ultrasound expert consultant. TS and CD do not own patents or intellectual property, nor work for or own stock and financial interests in any company in the neuromodulation field.

The remaining authors declare that the research was conducted in the absence of any commercial or financial relationships that could be construed as a potential conflict of interest.

## Footnotes

1 http://www.insight-journal.org/rire

## References

[B1] Ahearn E. P. E. (1997). The use of visual analog scales in mood disorders: a critical review. *J. Psychiatr. Res.* 31 569–579. 10.1016/S0022-3956(97)00029-0 9368198

[B2] Andrews-hanna J. R. Reidler J. S. Sepulcre J. Poulin R. Buckner R. L. (2010). Functional-anatomic fractionation of the brain’s default network. *Neuron* 65 550–562. 10.1016/j.neuron.2010.02.005 20188659 PMC2848443

[B3] Aron A. R. Robbins T. W. Poldrack R. A. (2014). Inhibition and the right inferior frontal cortex: one decade on. *Trends Cogn. Sci.* 18 177–185. 10.1016/j.tics.2013.12.003 24440116

[B4] Barnett S. B. Ter Haar G. R. Ziskin M. C. Rott H. D. Duck F. A. Maeda K. (2000). International recommendations and guidelines for the safe use of diagnostic ultrasound in medicine. *Ultrasound Med. Biol.* 26 355–366. 10.1016/S0301-5629(00)00204-0 10773365

[B5] Behzadi Y. Restom K. Liau J. Liu T. T. (2007). A component based noise correction method (CompCor) for BOLD and perfusion based fMRI. *Neuroimage* 37 90–101. 10.1016/j.neuroimage.2007.04.042 17560126 PMC2214855

[B6] Berkman E. T. Lieberman M. D. (2009). Using neuroscience to broaden emotion regulation: theoretical and methodological considerations. *Soc. Pers. Psychol. Compass* 3 475–493. 10.1111/j.1751-9004.2009.00186.x 24052803 PMC3775274

[B7] Bystritsky A. Korb A. S. (2015). A review of low-intensity transcranial focused ultrasound for clinical applications. *Curr. Behav. Neurosci. Rep.* 2 60–66. 10.1007/s40473-015-0039-0

[B8] Chapman I. V. MacNally N. A. Tucker S. (1980). Ultrasound-induced changes in rates of influx and efflux of potassium ions in rat thymocytes in vitro. *Ultrasound Med. Biol.* 6 47–49. 10.1016/0301-5629(80)90063-0 6245493

[B9] Chatrian G. E. Lettich E. Nelson P. L. (2018). Ten percent electrode system for topographic studies of spontaneous and evoked EEG activities. *Am. J. EEG Technol.* 25 83–92. 10.1080/00029238.1985.11080163

[B10] Chen Y. Wang C. Zhu X. Tan Y. Zhong Y. (2015). Aberrant connectivity within the default mode network in first-episode, treatment-naïve major depressive disorder. *J. Affect. Disord.* 183 49–56. 10.1016/j.jad.2015.04.052 26001663

[B11] Chiu P. H. Holmes A. J. Pizzagalli D. A. (2008). Dissociable recruitment of rostral anterior cingulate and inferior frontal cortex in emotional response inhibition. *Neuroimage* 42 988–997. 10.1016/j.neuroimage.2008.04.248 18556218 PMC2604817

[B12] Church C. C. Carstensen E. L. Nyborg W. L. Carson P. L. Frizzell L. A. Bailey M. R. (2008). The risk of exposure to diagnostic ultrasound in postnatal subjects: nonthermal mechanisms. *J. Ultrasound Med.* 27 565–592. 10.7863/jum.2008.27.4.565 18359909

[B13] Clasen P. C. Beevers C. G. Mumford J. A. Schnyer D. M. (2014). Cognitive control network connectivity in adolescent women with and without a parental history of depression. *Dev. Cogn. Neurosci.* 7 13–22. 10.1016/j.dcn.2013.10.008 24270043 PMC4209722

[B14] Coan J. A. Allen J. J. (2004). Frontal EEG asymmetry as a moderator and mediator of emotion. *Biol. Psychol.* 67 7–50. 10.1016/j.biopsycho.2004.03.002 15130524

[B15] Craig A. D. (2005). Forebrain emotional asymmetry: a neuroanatomical basis? *Trends Cogn. Sci.* 9 566–571. 10.1016/j.tics.2005.10.005 16275155

[B16] Dalecki D. (2004). Mechanical bioeffects of ultrasound. *Ann. Rev. Biomed. Eng.* 6 229–248. 10.1146/annurev.bioeng.6.040803.140126 15255769

[B17] Dallapiazza R. F. Timbie K. F. Holmberg S. Gatesman J. Lopes M. B. Price R. J. (2017). Noninvasive neuromodulation and thalamic mapping with low-intensity focused ultrasound. *J. Neurosurg.* 128 875–884. 10.3171/2016.11.JNS16976 28430035 PMC7032074

[B18] Davidson R. J. (2004). What does the prefrontal cortex “do” in affect: perspectives on frontal EEG asymmetry research. *Biol. Psychol.* 67 219–233. 10.1016/j.biopsycho.2004.03.008 15130532

[B19] Downs M. E. Buch A. Karakatsani M. E. Teichert T. Sierra C. Chen S. (2016). Focused ultrasound enhances decision-making in monkeys. *BioRxiv* [Preprint], 10.1101/041152

[B20] Drevets W. C. Price J. L. Furey M. L. (2008). Brain structural and functional abnormalities in mood disorders: implications for neurocircuitry models of depression. *Brain Struct. Funct.* 213 93–118. 10.1007/s00429-008-0189-x 18704495 PMC2522333

[B21] Fini M. Tyler W. J. (2017). Transcranial focused ultrasound: a new tool for non-invasive neuromodulation. *Intern. Rev. Psychiat.* 29 168–177. 10.1080/09540261.2017.1302924 28430535

[B22] Fitzgerald P. B. Laird A. R. Maller J. Daskalakis Z. J. (2008). A meta-analytic study of changes in brain activation in depression. *Hum. Brain Mapp.* 29 683–695. 10.1002/hbm.20426.A 17598168 PMC2873772

[B23] Folloni D. Verhagen L. Mars R. B. Fouragnan E. Constans C. Aubry J. F. (2019). Manipulation of subcortical and deep cortical activity in the primate brain using transcranial focused ultrasound stimulation. *Neuron* 101 1109–1116.e5. 10.1016/j.neuron.2019.01.019 30765166 PMC6520498

[B24] Fry W. (1957). Use of intense ultrasound in neurological research. *Am. J. Phys. Med.* 37 143–147.r 13545380

[B25] George M. S. Nahas Z. Molloy M. Speer A. M. Oliver N. C. Li X. B. (2000). A controlled trial of daily left prefrontal cortex TMS for treating depression. *Biol. Psychiatr.* 48 962–970. 10.1016/S0006-3223(00)01048-9 11082469

[B26] Goldin P. R. McRae K. Ramel W. Gross J. J. (2008). The neural bases of emotion regulation: reappraisal and suppression of negative emotion. *Biol. Psychiatr.* 63 577–586. 10.1016/j.biopsych.2007.05.031 17888411 PMC2483789

[B27] Golkar A. Lonsdorf T. B. Olsson A. Lindstrom K. M. Berrebi J. Fransson P. (2012). Distinct contributions of the dorsolateral prefrontal and orbitofrontal cortex during emotion regulation. *PLoS One* 7:48107. 10.1371/journal.pone.0048107 23144849 PMC3492343

[B28] Greicius M. D. Krasnow B. Reiss A. L. Menon V. (2003). Functional connectivity in the resting brain: a network analysis of the default mode hypothesis. *Proc. Natl. Acad. Sci. U.S.A.* 100 253–258. 10.1073/pnas.0135058100 12506194 PMC140943

[B29] Guo H. Hamilton M. Offutt S. J. Gloeckner C. D. Li T. Kim Y. (2018). Ultrasound produces extensive brain activation via a cochlear pathway. *Neuron* 98 1020–1030.e4. 10.1016/j.neuron.2018.04.036 29804919

[B30] Gusnard D. A. Akbudak E. Shulman G. L. Raichle M. E. (2001). Medial prefrontal cortex and self-referential mental activity: relation to a default mode of brain function. *Proc. Natl. Acad. Sci. U.S.A.* 98 4259–4264. 10.1073/pnas.071043098 11259662 PMC31213

[B31] Hameroff S. Penrose R. (2014). Consciousness in the universe a review of the ‘ Orch OR ’ theory. *Phys. Life Rev.* 11 39–78. 10.1016/j.plrev.2013.08.002 24070914

[B32] Hameroff S. Trakas M. Duffield C. Annabi E. Gerace M. B. Boyle P. (2013). Transcranial ultrasound (TUS) effects on mental states: a pilot study. *Brain Stimul.* 6 409–415. 10.1016/j.brs.2012.05.002 22664271

[B33] Hauptman J. S. DeSalles A. A. Espinoza R. Sedrak M. Ishida W. (2008). Potential surgical targets for deep brain stimulation in treatment-resistant depression. *Neurosurg. Focus* 25:E3. 10.3171/FOC/2008/25/7/E3 18590380

[B34] Kaiser R. H. Andrews-Hanna J. R. Wager T. D. Pizzagalli D. A. (2015). Large-scale network dysfunction in major depressive disorder: a meta-analysis of resting-state functional connectivity. *JAMA Psychiatry* 72 603–611. 10.1001/jamapsychiatry.2015.0071 25785575 PMC4456260

[B35] Kaiser R. H. Whitfield-Gabrieli S. Dillon D. G. Goer F. Beltzer M. Minkel J. (2016). Dynamic resting-state functional connectivity in major depression. *Neuropsychopharmacology* 41 1822–1830. 10.1038/npp.2015.352 26632990 PMC4869051

[B36] Kalisch R. (2009). The functional neuroanatomy of reappraisal: time matters. *Neurosci. Biobehav. Rev.* 33 1215–1226. 10.1016/j.neubiorev.2009.06.003 19539645

[B37] Keng S. L. Smoski M. J. Robins C. J. (2011). Effects of mindfulness on psychological health: a review of empirical studies. *Clin. Psychol. Rev.* 31 1041–1056. 10.1016/j.cpr.2011.04.006 21802619 PMC3679190

[B38] Kerestes R. Bhagwagar Z. Nathan P. J. Meda S. A. Ladouceur C. D. Maloney K. (2012). Prefrontal cortical response to emotional faces in individuals with major depressive disorder in remission. *Psychiatry Res. Neuroimag.* 202 30–37. 10.1016/j.pscychresns.2011.11.004 22595508 PMC3995357

[B39] Killingsworth M. A. Gilbert D. T. (2010). A wandering mind is an unhappy mind. *Science* 12:932. 10.1126/science.1192439 21071660

[B40] Kim H. Lee S. D. Chiu A. Yoo S. S. Park S. (2014). Estimation of the spatial profile of neuromodulation and the temporal latency in motor responses induced by focused ultrasound brain stimulation. *Neuroreport* 25 475–479. 10.1097/WNR.0000000000000118 24384503 PMC3979873

[B41] Klem G. H. Lüders H. O. Jasper H. H. Elger C. (1999). The ten-twenty electrode system of the international federation. *Electroencephalogr. Clin. Neurophysiol.* 52 3–6.r 10590970

[B42] Koessler L. Maillard L. Benhadid A. Vignal J. P. Felblinger J. Vespignani H. (2009). Automated cortical projection of EEG sensors: anatomical correlation via the international 10-10 system. *Neuroimage* 46 64–72. 10.1016/j.neuroimage.2009.02.006 19233295

[B43] Krasovitski B. Frenkel V. Shoham S. Kimmel E. (2011). Intramembrane cavitation as a unifying mechanism for ultrasound-induced bioeffects. *Proc. Natl. Acad. Sci. U.S.A.* 108 3258–3263. 10.1073/pnas.1015771108 21300891 PMC3044354

[B44] Kubanek J. (2018). Neuromodulation with transcranial focused ultrasound. *Neurosurg. Focus* 44:E14. 10.3171/2017.11.FOCUS17621 29385924 PMC5927579

[B45] Kubanek J. Shi J. Marsh J. Chen D. Deng C. Cui J. (2016). Ultrasound modulates ion channel currents. *Sci. Rep.* 6 1–14. 10.1038/srep24170 27112990 PMC4845013

[B46] Lee W. Chung Y. A. Jung Y. Song I.-U. Yoo S.-S. (2016a). Simultaneous acoustic stimulation of human primary and secondary somatosensory cortices using transcranial focused ultrasound. *BMC Neurosci.* 17:68. 10.1186/s12868-016-0303-6 27784293 PMC5081675

[B47] Lee W. Kim H.-C. Jung Y. Chung Y. A. Song I.-U. Lee J.-H. (2016b). Transcranial focused ultrasound stimulation of human primary visual cortex. *Sci. Rep.* 6:34026. 10.1038/srep34026 27658372 PMC5034307

[B48] Lee W. Kim H. Jung Y. Song I.-U. Chung Y. A. Yoo S.-S. (2015). Image-guided transcranial focused ultrasound stimulates human primary somatosensory cortex. *Sci. Rep.* 5:8743. 10.1038/srep08743 25735418 PMC4348665

[B49] Lee W. Lee S. D. Park M. Y. Foley L. Purcell-Estabrook E. Kim H. (2016c). Image-guided focused ultrasound-mediated regional brain stimulation in sheep. *Ultrasound Med. Biol.* 42 459–470. 10.1016/j.ultrasmedbio.2015.10.001 26525652

[B50] Legon W. Ai L. Bansal P. Mueller J. K. (2018). Neuromodulation with single-element transcranial focused ultrasound in human thalamus. *Hum. Brain Mapp* 39 1995–2006. 10.1002/hbm.23981 29380485 PMC6866487

[B51] Legon W. Rowlands A. Opitz A. Sato T. F. Tyler W. J. (2012). Pulsed ultrasound differentially stimulates somatosensory circuits in humans as indicated by EEG and fMRI. *PLoS One* 7:e51177. 10.1371/journal.pone.0051177 23226567 PMC3514181

[B52] Legon W. Sato T. F. Opitz A. Mueller J. Barbour A. Williams A. (2014). Transcranial focused ultrasound modulates the activity of primary somatosensory cortex in humans. *Nat. Neurosci.* 17 322–329. 10.1038/nn.3620 24413698

[B53] Lindquist K. A. Satpute A. B. Wager T. D. Weber J. Barrett L. F. (2016). The brain basis of positive and negative affect: evidence from a meta-analysis of the human neuroimaging literature. *Cereb. Cortex* 26 1910–1922. 10.1093/cercor/bhv001 25631056 PMC4830281

[B54] Lozano A. M. Mayberg H. S. Craddock R. C. Kennedy S. H. (2010). Stimulation for treatment- resistant depression. *J. Am. Psychiatry* 8 583–591.

[B55] Marsh-Richard D. M. Hatzis E. S. Mathias C. W. Venditti N. Dougherty D. M. (2009). Adaptive Visual analog scales (AVAS): a modifiable software program for the creation, administration, and scoring of visual analog scales. *Behav. Res. Methods* 41 99–106. 10.3758/BRM.41.1.99 19182128 PMC2635491

[B56] Michael N. Erfurth A. (2004). Treatment of bipolar mania with right prefrontal rapid transcranial magnetic stimulation. *J. Affect. Disord.* 78 253–257. 10.1016/S0165-0327(02)00308-7 15013251

[B57] Monk T. H. (1989). A visual analogue scale technique to measure global vigor and affect. *Psychiatry Res.* 27 89–99. 10.1016/0165-1781(89)90013-9 2922449

[B58] Monti M. M. Schnakers C. Korb A. S. Bystritsky A. Vespa P. M. (2016). Non-invasive ultrasonic thalamic stimulation in disorders of consciousness after severe brain injury: a first-in-man report. *Brain Stimul.* 9 940–941. 10.1016/j.brs.2016.07.008 27567470

[B59] Mueller J. Legon W. Opitz A. Sato T. F. Tyler W. J. (2014). Transcranial focused ultrasound modulates intrinsic and evoked EEG dynamics. *Brain Stimul.* 7 900–908. 10.1016/j.brs.2014.08.008 25265863

[B60] Nyenhuis D. L. (1997). Standardization and validation of the standardization and validation of the visual analog mood scales. *Clin. Neuropsychol.* 11 407–415. 10.1080/13854049708400470 17394228

[B61] O’Brien W. D. (2007). Ultrasound-biophysics mechanisms. *Prog. Biophys. Mol. Biol.* 93 212–255. 10.1016/j.pbiomolbio.2006.07.010 16934858 PMC1995002

[B62] Ochsner K. N. Gross J. J. (2005). The cognitive control of emotion. *Trends Cogn. Sci.* 9 242–249. 10.1016/j.tics.2005.03.010 15866151

[B63] Parvaz M. A. MacNamara A. Goldstein R. Z. Hajcak G. (2012). Event-related induced frontal alpha as a marker of lateral prefrontal cortex activation during cognitive reappraisal. *Cogn. Affect. Behav. Neurosci.* 12 730–740. 10.3758/s13415-012-0107-9 22773414 PMC3494774

[B64] Phan K. L. Wager T. Taylor S. F. Liberzon I. (2002). Functional neuroanatomy of emotion: a meta-analysis of emotion activation studies in PET and fMRI. *Neuroimage* 16 331–348. 10.1006/nimg.2002.1087 12030820

[B65] Phillips M. L. Drevets W. C. Rauch S. L. Lane R. (2003). Neurobiology of emotion perception I: the neural basis of normal emotion perception. *Biol. Psychiatry* 54 504–514. 10.1016/S0006-3223(03)00168-9 12946879

[B66] Phillips M. L. Ladouceur C. D. Drevets W. C. (2008). A neural model of voluntary and automatic emotion regulation: implications for understanding the pathophysiology and neurodevelopment of bipolar disorder. *Mol. Psychiatry* 13 833–857. 10.1038/mp.2008.65 18574483 PMC2745893

[B67] Price J. L. Drevets W. C. (2012). Neural circuits underlying the pathophysiology of mood disorders. *Trends Cogn. Sci.* 16 61–71. 10.1016/j.tics.2011.12.011 22197477

[B68] Ray R. D. Zald D. H. (2012). Anatomical insights into the interaction of emotion and cognition in the prefrontal cortex. *Neurosci. Biobehav. Rev.* 36 479–501. 10.1016/j.neubiorev.2011.08.005 21889953 PMC3244208

[B69] Rempel-Clower N. L. (2007). Role of orbitofrontal cortex connections in emotion. *Ann. N. Y. Acad. Sci. U.S.A.* 1121 72–86. 10.1196/annals.1401.026 17846152

[B70] Renner F. Siep N. Arntz A. van de Ven V. Peeters F. P. M. L. Quaedflieg C. W. E. M. (2017). Negative mood-induction modulates default mode network resting-state functional connectivity in chronic depression. *J. Affect. Disord.* 208 590–596. 10.1016/j.jad.2016.10.022 27810271

[B71] Riva P. Romero Lauro L. J. DeWall C. N. Bushman B. J. (2012). Buffer the pain away: stimulating the right ventrolateral prefrontal cortex reduces pain following social exclusion. *Psychol. Sci.* 23 1473–1475. 10.1177/0956797612450894 23132013

[B72] Riva P. Romero Lauro L. J. DeWall C. N. Chester D. S. Bushman B. J. (2015a). Reducing aggressive responses to social exclusion using transcranial direct current stimulation. *Soc. Cogn. Affect. Neurosci.* 10 352–356. 10.1093/scan/nsu053 24748546 PMC4350477

[B73] Riva P. Romero Lauro L. J. Vergallito A. DeWall C. N. Bushman B. J. (2015b). Electrified emotions: modulatory effects of transcranial direct stimulation on negative emotional reactions to social exclusion. *Soc. Neurosci.* 10 46–54. 10.1080/17470919.2014.946621 25139575

[B74] Sahu S. Ghosh S. Ghosh B. Aswani K. Hirata K. Fujita D. (2013). Atomic water channel controlling remarkable properties of a single brain microtubule: correlating single protein to its supramolecular assembly. *Biosens. Bioelectron.* 47 141–148. 10.1016/j.bios.2013.02.050 23567633

[B75] Sang H. K. Hamann S. (2007). Neural correlates of positive and negative emotion regulation. *J. Cogn. Neurosci.* 19 776–798. 10.1162/jocn.2007.19.5.776 17488204

[B76] Sanguinetti J. L. Smith E. E. Dieckman L. Vanuk J. Hameroff S. Allen J. J. B. (2013). Transcranial ultrasound for brain stimulation: effects on mood. *Psychophysiology* 50:S46.r

[B77] Sassaroli E. Vykhodtseva N. (2016). Acoustic neuromodulation from a basic science prospective. *J. Therap. Ultrasound* 4 1–14. 10.1186/s40349-016-0061-z 27213044 PMC4875658

[B78] Sato T. Shapiro M. G. Tsao D. Y. (2018). Ultrasonic neuromodulation causes widespread cortical activation via an indirect auditory mechanism. *Neuron* 98 1031–1041.e5. 10.1016/j.neuron.2018.05.009 29804920 PMC8127805

[B79] Sheline Y. I. Barch D. M. Price J. L. Rundle M. M. Vaishnavi S. N. Snyder A. Z. (2009). The default mode network and self-referential processes in depression. *Proc. Natl. Acad. Sci. U.S.A.* 106 1942–1947. 10.1073/pnas.0812686106 19171889 PMC2631078

[B80] Stewart J. L. Coan J. A. Towers D. N. Allen J. J. B. (2014). Resting and task-elicited prefrontal EEG alpha asymmetry in depression: support for the capability model. *Psychophysiology* 51 446–455. 10.1111/psyp.12191 24611480 PMC3984363

[B81] Taylor V. A. Daneault V. Grant J. Scavone G. Breton E. Roffe-vidal S. (2013). Impact of meditation training on the default mode network during a restful state. *Soc. Cogn. Affect. Neurosci.* 8 4–14. 10.1093/scan/nsr087 22446298 PMC3541485

[B82] ter Haar G. (2007). Therapeutic applications of ultrasound. *Prog. Biophys. Mol. Biol.* 93 111–129. 10.1016/j.pbiomolbio.2006.07.005 16930682

[B83] Touroutoglou A. Lindquist K. A. Dickerson B. C. Barrett L. F. (2014). Intrinsic connectivity in the human brain does not reveal networks for “basic” emotions. *Soc. Cogn. Affect. Neurosci.* 10 1257–1265. 10.1093/scan/nsv013 25680990 PMC4560947

[B84] Treeby B. E. Cox B. T. (2010). k-Wave: MATLAB toolbox for the simulation and reconstruction of photoacoustic wave fields. *J. Biomed. Opt.* 15:021314. 10.1117/1.3360308 20459236

[B85] Tufail Y. Matyushov A. Baldwin N. Tauchmann M. L. Georges J. Yoshihiro A. (2010). Transcranial pulsed ultrasound stimulates intact brain circuits. *Neuron* 66 681–694. 10.1016/j.neuron.2010.05.008 20547127

[B86] Tyler W. J. (2011). Noninvasive neuromodulation with ultrasound? A continuum mechanics hypothesis. *Neuroscientist* 17 25–36. 10.1177/1073858409348066 20103504

[B87] Tyler W. J. Tufail Y. Finsterwald M. Tauchmann M. L. Olson E. J. Majestic C. (2008). Remote excitation of neuronal circuits using low-intensity, low-frequency ultrasound. *PLoS One* 3:3511. 10.1371/journal.pone.0003511 18958151 PMC2568804

[B88] Vergallito A. Riva P. Pisoni A. Lauro L. J. R. (2018). Modulation of negative emotions through anodal tDCS over the right ventrolateral prefrontal cortex. *Neuropsychologia* 119 128–135. 10.1016/j.neuropsychologia.2018.07.037 30089234

[B89] Verhagen L. Gallea C. Folloni D. Constans C. Jensen D. E. A. Ahnine H. (2019). Offline impact of transcranial focused ultrasound on cortical activation in primates. *eLife* 8:e40541. 10.7554/eLife.40541 30747105 PMC6372282

[B90] Wager T. D. Davidson M. L. Hughes B. L. Lindquist M. A. Ochsner K. N. (2008). Prefrontal-subcortical pathways mediating successful emotion regulation. *Neuron* 59 1037–1050. 10.1016/j.neuron.2008.09.006 18817740 PMC2742320

[B91] Whitfield-Gabrieli S. Nieto-Castanon A. (2012). Conn: a functional connectivity toolbox for correlated and anticorrelated brain networks. *Brain Connect.* 2 125–141. 10.1089/brain.2012.0073 22642651

[B92] Wu J. Nyborg W. L. (2008). Ultrasound, cavitation bubbles and their interaction with cells. *Adv. Drug Deliv. Rev.* 60 1103–1116. 10.1016/j.addr.2008.03.009 18468716

[B93] Yoo S.-S. Bystritsky A. Lee J.-H. Zhang Y. Fischer K. Min B.-K. (2011). Focused ultrasound modulates region-specific brain activity. *Neuroimage* 56 1267–1275. 10.1016/j.neuroimage.2011.02.058 21354315 PMC3342684

