# Supplementary material for: Transcranial Focused Ultrasound to the Right Prefrontal Cortex Improves Mood and Alters Functional Connectivity in Humans
Source: Front Hum Neurosci. 2020 Feb 28;14:52. doi: 10.3389/fnhum.2020.00052 (PMC7058635; doi:10.3389/fnhum.2020.00052)
Supplement: Supplementary file 1 [file Table_1.docx]

Supplemental Figure 1. The Global Affect scores from the VAMS scale for each subject who *did not* report hearing a sound (Means: Baseline: 71; Post-10: 78; Post-20: 81; Post-30: 82).

Supplemental Figure 2. The Global Affect scores from the VAMS scale for each subject who *did* report hearing a sound (Means: Baseline: 69; Post-10: 65; Post-20: 72; Post-30: 71).

Supplemental Figure 3. The Happiness question from the VAMS scale. Standard error of the mean is shown on the graph. Happiness, Calm, Sad, and Tense subscales are combined to create the Global Affect measure.

Supplemental Figure 4. The Calm question from the VAMS scale. Standard error of the mean is shown on the graph.

Supplemental Figure 5. The Sad question from the VAMS scale. Standard error of the mean is shown on the graph.

Supplemental Figure 6. The Tense question from the VAMS scale. Standard error of the mean is shown on the graph.

Supplemental Figure 7. The Effort question from the VAMS scale. Standard error of the mean is shown on the graph. Effort, Sleepy, Weary, and Alert are used to create the Global Vigor question from the VAMS scales.

Supplemental Figure 8. The Sleepy question from the VAMS scale. Standard error of the mean is shown on the graph.

Supplemental Figure 9. The Weary question from the VAMS scale. Standard error of the mean is shown on the graph.

Supplemental Figure 10. The Alert question from the VAMS scale. Standard error of the mean is shown on the graph.
